# Retraction: Vascular Endothelial Growth Factor Receptor-1 Activation Promotes Migration and Invasion of Breast Cancer Cells through Epithelial-Mesenchymal Transition

**DOI:** 10.1371/journal.pone.0274216

**Published:** 2022-08-31

**Authors:** The PLOS ONE Editors

Following the publication of this article [1], concerns were raised regarding results presented in multiple figures. Specifically,

- In Figure 6A, tumor volume appears to exceed internationally-accepted standards, raising concern about animal welfare and the adherence of the study to animal research ethics standards.
- The shRNA and shRNA + PIGF panels for MDA-MB-231 in Figure 3B and the PIGF panel for MDA-MB-231 in Figure 3C appear to partially overlap despite representing different conditions.

- The following panels appear similar despite representing different conditions:
  - The β-actin panel for MDA-MB-231 in Figure 3A and lanes 1–3 of the E-cadherin panel in Fig 4D with the aspect ratio altered.
  - The β-catenin panel in Figure 4E and the Snail (6 hours) panel for MDA-MB-231 in Figure 5.
- The following loading control panels appear similar:
  - Lamin B1 in Figure 4D and Lamin B1 for MCF-7 in Figure 5.
  - Lamin B1 in Figure 4E and Lamin B1 for MDA-MB-231 in Figure 5.
- No sequence is provided in the article for the control shRNA. The corresponding author acknowledged that they no longer have a record of the control shRNA sequence.

The corresponding author stated that mice were observed every day for mental state, food intake, activity, and diameter of the tumor at the injection site. They confirmed that after 30 days, half the mice had lost weight and were exhibiting behavioral changes, and at that point, the experiment was terminated. Data files described as raw tumor and body weight data were provided which indicated that weight loss was observed from day 14 onwards and final tumor weight exceeded 10% body weight in some mice. Concerns regarding tumor volume and animal welfare indicate that the article is not in compliance with PLOS’s Animal Research policy.

The corresponding author provided raw image data underlying the western blots in Figures 2A, 3A, 4D, 4E, and 5. They acknowledged that the following panels are incorrect and provided replacement data:

- The shRNA + PIGF panel for MDA-MB-231 in Figure 3B and the PIGF panel for MDA-MB-231 in Figure 3C.
- The β-actin panel for MDA-MB-231 in Figure 3A.
- The Snail (6 hours) panel for MDA-MB-231 in Figure 5.

In the raw image provided for the Snail (6 hours) panel for MCF-7 in Figure 5, a rectangular area unlike the rest of the background is observed in the shRNA + PIGF lane when color levels are adjusted. The Editors remain concerned about the integrity of this raw image.

The corresponding author stated that Lamin B1 loading control was measured for each of the western blot experiments in Figures 4D, 4E, and 5, but that the same Lamin B1 images were reused in preparation of the figures. The correct loading control images corresponding to each experiment were not provided.

In light of the above concerns regarding animal welfare which indicate that the article is not in compliance with PLOS’s Animal Research policy, and the concerns affecting images in multiple figures which question the reliability of these data, the *PLOS ONE* Editors retract this article.

XZhao did not agree with the retraction and apologizes for the issues with the published article. CL responded but expressed neither agreement nor disagreement with the editorial decision. QN, LH, MM, XZhang, ML, SS, and XZuo either did not respond directly or could not be reached.
